# Macrophage activation syndrome with acute hepatitis in a patient with adult-onset immunodeficiency with anti-interferon gamma antibodies: a case report

**DOI:** 10.1186/s13256-023-04245-w

**Published:** 2024-01-05

**Authors:** William Hirsch, Bryant Megna, Oyedele Adeyi, Nicholas Lim

**Affiliations:** 1 Department of Medicine, University of Minnesota, Minneapolis, MN USA; 2 Division of Gastroenterology, Hepatology, & Nutrition, Department of Medicine, University of Minnesota, Minneapolis, MN USA; 3 Department of Laboratory Medicine & Pathology, University of Minnesota, Minneapolis, MN USA

**Keywords:** Macrophage activation syndrome, Adult-onset immunodeficiency with anti-interferon gamma antibodies, Acute hepatitis, Liver biopsy

## Abstract

**Background:**

Macrophage activation syndrome is a rare disorder leading to unregulated immune activity manifesting with nonspecific constitutional symptoms, laboratory abnormalities, and multiorgan involvement. We report the case of a patient who presented with acute hepatitis secondary to macrophage activation syndrome diagnosed by liver biopsy and successfully treated with intravenous immune globulin, anakinra, and rituximab.

**Case presentation:**

A 42-year-old Laotian woman with adult-onset immunodeficiency with anti-interferon gamma antibodies presented with a fever, headache, generalized myalgia, dark urine, and reduced appetite in the setting of family members at home with similar symptoms. Her laboratory workup was notable for evidence of acute hepatitis without acute liver failure. After an unrevealing comprehensive infectious and noninvasive rheumatologic workup was completed, a liver biopsy was performed ultimately revealing the diagnosis of macrophage activation syndrome. She was successfully treated with intravenous immune globulin, anakinra, and rituximab.

**Conclusion:**

This case highlights the importance of maintaining macrophage activation syndrome on the differential of a patient with acute hepatitis of unknown etiology in the correct clinical context and the value of a liver biopsy in making a diagnosis when noninvasive testing is unrevealing.

## Background

Macrophage activation syndrome (MAS) is thought to be related to a defect in lymphocyte cytolytic activity responsible for maintaining appropriate level of immune response to tissue injury from infection or inflammation [1]. MAS can be a challenging diagnosis to make given its nonspecific clinical manifestations, most commonly featuring fever, hyperferritinemia, hypertriglyceridemia, pancytopenia, fibrinolytic consumptive coagulopathy, and hepatic dysfunction [1]. Several case reports have characterized various presentations of MAS with a common theme of underlying immunodeficiency secondary to an autoimmune condition triggered by a superimposed infection [2–7]. We report the case of a patient with adult-onset immunodeficiency with anti-interferon gamma antibodies who presented with acute hepatitis ultimately diagnosed with MAS through liver biopsy.

## Case presentation

Two months prior to admission to our hospital, a 42-year-old Laotian woman was hospitalized for an expedited workup of a constellation of symptoms, including progressive generalized musculoskeletal pain, persistent dry cough, weakness, generalized lymphadenopathy, and unintentional 20-pound weight loss. During that admission, she was diagnosed with disseminated *Mycobacterium hassicum* infection and started on a 6-month course of clarithromycin, imipenem/cilastatin, moxifloxacin, and ceftaroline. Because this opportunistic infection is typically seen in immunocompromised individuals, a workup to determine the underlying cause of her immunodeficient status was initiated and revealed adult-onset immunodeficiency with anti-interferon gamma antibodies.

Two months after initiation of her antibiotic regimen, the patient represented for evaluation of fever, headache, generalized myalgia, dark urine, and reduced appetite in the setting of family members at home with similar symptoms. Her vital signs were notable for fever to 102.9 ℉ and tachycardia. Initial laboratories were notable for a respiratory viral panel positive for human rhinovirus and a hepatic panel showing elevated aspartate aminotransferase (AST) 1281 U/L (ref < 45 U/L), alanine aminotransferase (ALT) 511 U/L (ref < 50 U/L), and alkaline phosphatase (ALP) 265 U/L (ref 40–150 U/L) (Table 1). The patient had no history of liver disease and liver function tests were normal as recently as 2 weeks prior to this admission. Her total bilirubin, direct bilirubin, and ammonia were within normal limits, and international normalized ratio (INR) was elevated at 1.60 (ref 0.85–1.15). Viral hepatitis panel (including hepatitis A, B, C and HIV), abdominal ultrasound with Doppler, and autoimmune hepatitis serologies (including antismooth muscle, antinuclear, and antimitochondrial antibodies) were unrevealing.

**Table 1:** Notable laboratory values during hospitalization

	Patient’s result day 1	Patient’s result day 5	Patient’s result day 14	Patient’s result day 90	Reference range
Total bilirubin (TBili) mg/dL	1.2	1.3	0.7	0.9	0.2–1.3
Aspartate aminotransferase (AST) (U/L)	1281	4822	28	17	< 45
Alanine aminotransferase (ALT) (U/L)	511	686	55	27	< 50
Alkaline phosphatase (ALP) (U/L)	265	632	194	57	40–150
INR	1.60	1.31	1.18		0.85–1.15
Ferritin (ng/mL)		62,225	659	25	12–150
Triglycerides (ng/dL)		343			< 150
C-reactive protein (mg/L)		16.2	< 3.0	< 3.0	< 5
Hemoglobin (g/dL)	8.9	9.2	8.0	11.2	11.7–15.7
White blood count (10^3^ L)	5.3	20.3	5.8	3.6	4.0–11.0
Platelet count (10^3^/μL)	101	123	192	236	150–450

*AST* Aspartate aminotransferase; *ALT* Alanine aminotransferase; *ALP* Alkaline phosphatase; *INR* International normalized ratio; *CRP* C-reactive protein

The patient’s liver enzymes continued to rise over the next several days to AST 4188 U/L, ALT 684 U/L, and ALP 669 U/L with no corresponding decline in synthetic liver function. After consultation with infectious disease specialists, the patient’s antibiotic regimen was held due to concern for possible acute hepatitis secondary to drug-induced liver injury. During this time, she remained intermittently febrile and developed a generalized tonic–clonic seizure. Subsequent nuchal rigidity prompted a lumbar puncture and empiric treatment for meningitis. A comprehensive rheumatologic and infectious disease workup had been unrevealing to this point. With new infections and malignancy essentially ruled out, work up for a dysregulated immune response was pursued, revealing a ferritin level of 62,225 ng/mL (ref 6–175 ng/mL), triglycerides 343 mg/dL (ref  < 150 mg/dL), white blood cell count 12,600/μL (ref 4000–11,000/μL), hemoglobin 8.4 g/dL (ref 11.7–15.7 g/dL), and platelet count 27,000/μL (ref 150,000–450,000/μL). Notably, a soluble interleukin (IL) 2 receptor alpha level resulted 6477 pg/mL (ref < 2700 pg/mL).

**Fig. 1:**
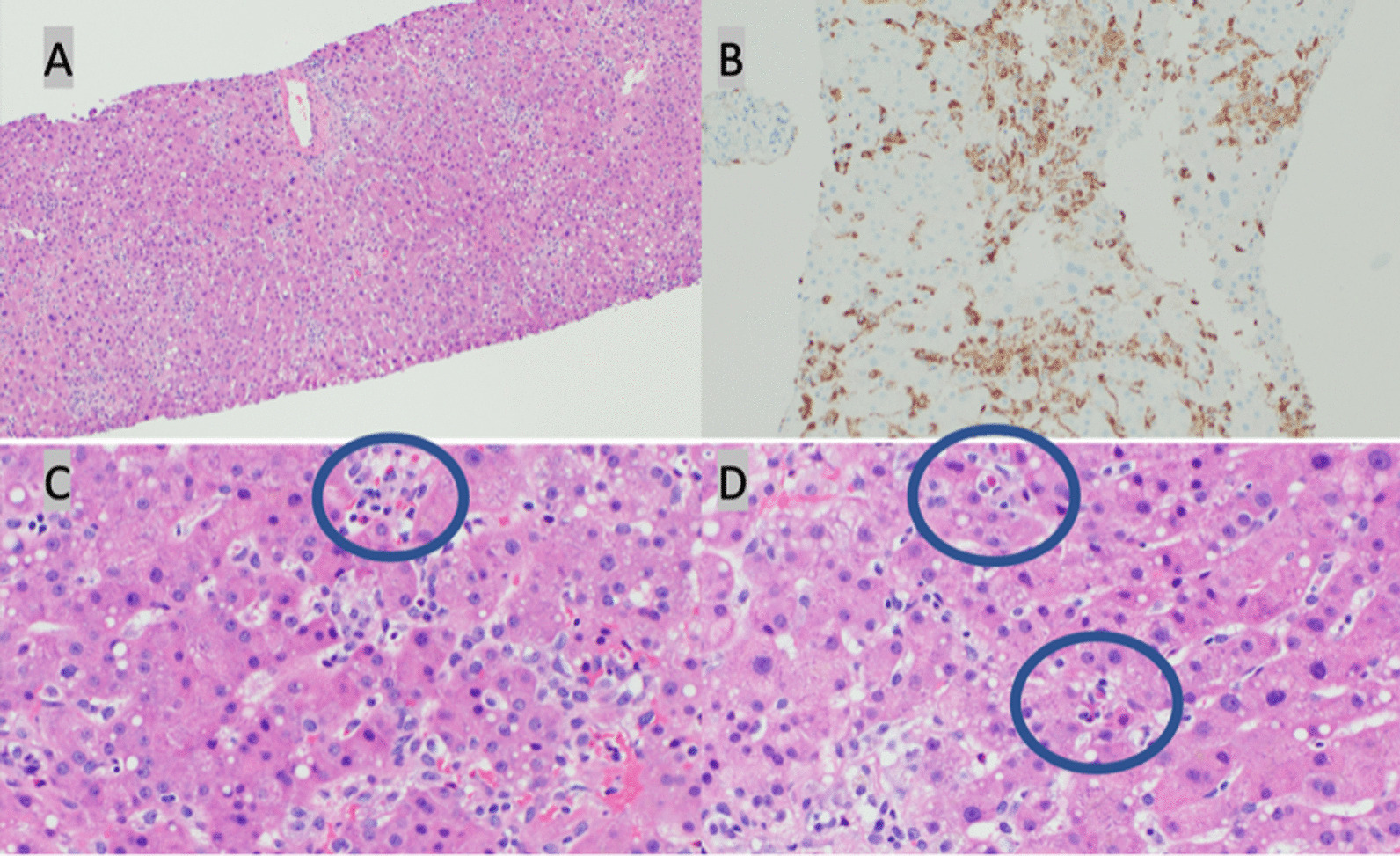
**A** Hematoxylin and eosin (H&E) 100 ×: clusters of sinusoidal histiocytes. **B** CD68 stain, 200 ×: clusters of sinusoidal histiocytes. **C** H&E 400 ×: clusters of sinusoidal histiocytes with hemophagocytosis (circle). **D** H&E 400 ×: clusters of sinusoidal histiocytes with hemophagocytosis (circles)

A liver biopsy was obtained to better characterize the systemic process affecting the patient. The biopsy revealed prominent predominantly sinusoidal but also lobar and portal clusters of reactive Kupffer cells with consequent small foci of hepatocellular injury and necrosis (Fig. 1). Bile duct architecture was preserved and immunostains were negative for adenovirus, cytomegalovirus, acid fast bacilli, and Epstein–Barr virus. With the patient’s history of acquired immunodeficiency and clinical presentation, the liver biopsy findings supported a diagnosis of acute hepatitis secondary to dysregulated immune response to rhinovirus infection leading to MAS. Treatment with intravenous immune globulin (IVIG), anakinra, and rituximab was initiated by consultants from hematology and rheumatology with subsequent improvement in clinical status, liver function tests, platelet count, and ferritin. Liver function tests improved within 2 weeks and normalized within 3 months. At most recent follow-up, the patient reported feeling well with resolution of presenting symptoms and normalization in inflammatory markers.

## Discussion and conclusion

This case presents an opportunity to explore a rare diagnosis of MAS presenting as acute hepatitis in a patient with a rare underlying etiology of immunodeficiency. Our patient was diagnosed with acquired immunodeficiency with anti-interferon gamma autoantibodies during her workup for nontuberculous mycobacterial infection prior to her presentation at our hospital. Acquired immunodeficiency with anti-interferon gamma autoantibodies is characterized by profound immunodeficiency with poor response to appropriate therapy for recurrent opportunistic infections [8]. Anti-interferon autoantibodies have previously been described to be frequently detected in Asian adults presenting with multiple opportunistic infections [9]. The cause of the development in these autoantibodies remains unknown; however, it is suspected to be related to incompletely understood genetic factors [8]. While MAS is seen in the context of autoimmunity, we are unaware of any previously described cases of the development of MAS in a patient with acquired immunodeficiency with anti-interferon gamma autoantibodies [10].

There is a significant overlap between the presence of autoimmune and autoinflammatory diseases and the development of MAS [11]. This is notable given our patient’s history of immunodeficiency secondary to acquired anti-interferon gamma antibodies. This underlying immunodeficiency likely served as the catalyst for a dysregulated immune response to an otherwise uneventful rhinovirus infection. There are several known etiologies of MAS, including triggers such as infection, inflammation, and malignancy. These triggers can lead to dysregulated activation of IL-1 and IL-18 pathways [10]. The pathophysiology of local hepatic tissue inflammation and destruction secondary to Kupffer cell activation has been previously well-described [12]. One case series of pediatric patients with MAS demonstrated liver biopsies with interferon gamma (IFN-γ) expression by CD8^+^ lymphocytes and IL-6 and tumor necrosis factor alpha (TNF-α) expression by hemophagocytosing macrophages [13]. These findings support the rationale behind the treatment strategy implemented for our patient including IVIG and the IL-1 receptor antagonist, anakinra. In general, treatment for MAS typically combines some form of IL-1 suppression, corticosteroids, and the calcineurin inhibitor, cyclosporine [14]. Without early diagnosis and intervention, MAS has a high fatality rate [15].

Even with several prototypical features of MAS present in our patient, the diagnosis of MAS was elusive until a thoroughly negative infectious and noninvasive rheumatologic workup had been completed and the diagnosis not clinched until the results of the liver biopsy returned. A recent review demonstrated that liver biopsy successfully leads to a specific diagnosis in 84% of patients with persistently abnormal liver enzymes of unclear etiology [16]. Noninvasive imaging has recently increased in popularity owing to its accessibility and cost benefits, while competence in liver biopsy is no longer a requirement for advanced hepatology trainees [17]. However, liver biopsy remains irreplaceable in the diagnosis of infiltrative diseases and can offer a definitive diagnosis when the etiology of abnormal liver enzyme levels is unknown [18, 19]. In cases where understanding the etiology of acute hepatitis will ultimately guide disease course-modifying, and potentially life-saving, therapy, the benefits of liver biopsy far outweigh the associated risks; a recent review quantified the overall rate of bleeding after image-guided liver biopsy as 2%[20].

In summary, MAS is an inflammatory condition characterized by fever, lymphadenopathy, hepatosplenomegaly, pancytopenia, coagulopathy, and multiorgan involvement. This case highlights the importance of maintaining MAS on the differential diagnosis when considering a patient with the above constellation of symptoms, laboratory results, and physical exam findings. It also highlights the value of a liver biopsy in confirming the diagnosis and guiding targeted therapy in the setting of hepatic involvement.

## Data Availability

Not applicable.

## Abbreviations

MAS: Macrophage activation syndrome
AST: Aspartate aminotransferase
ALT: Alanine aminotransferase
ALP: Alkaline phosphatase
INR: International normalized ratio
TBili: Total bilirubin
IL: Interleukin
IVIG: Intravenous immune globulin
TNF-α: Tumor necrosis factor alpha
IFN-γ: Interferon gamma

## References

[1] Crayne CB, Albeituni S, Nichols KE, Cron RQ (2019). The immunology of macrophage activation syndrome. Front Immunol.

[2] Jindal AK, Agarwal A, Guleria S (2018). Adult-onset still disease and macrophage activation syndrome following Chikungunya and Hepatitis E Coinfection. J Clin Rheumatol Pract Rep Rheum Musculoskelet Dis.

[3] Nagel M, Schwarting A, Straub BK, Galle PR, Zimmermann T (2017). Hepatic manifestation of a macrophage activation syndrome (MAS). Z Gastroenterol.

[4] Kumar A, Kato H (2016). Macrophage activation syndrome associated with adult-onset still’s disease successfully treated with anakinra. Case Rep Rheumatol.

[5] Casciaro R, Cresta F, Favilli F, Naselli A, De Alessandri A, Minicucci L (2014). Macrophage activation syndrome induced by A/H1N1 influenza in cystic fibrosis. Pediatr Pulmonol.

[6] Russo RAG, Rosenzweig SD, Katsicas MM (2008). Hepatitis A-associated macrophage activation syndrome in children with systemic juvenile idiopathic arthritis: report of 2 cases. J Rheumatol.

[7] AlNouwaiser DN, AlMutairi SS, AlRowailey AS (2022). Management of hepatitis a-induced macrophage activation syndrome with anakinra in systemic juvenile idiopathic arthritis: a case report. Cureus.

[8] Pithukpakorn M, Roothumnong E, Angkasekwinai N (2015). HLA-DRB1 and HLA-DQB1 are associated with adult-onset immunodeficiency with acquired anti-interferon-gamma autoantibodies. PLoS ONE.

[9] Browne SK, Burbelo PD, Chetchotisakd P (2012). Adult-onset immunodeficiency in Thailand and Taiwan. N Engl J Med.

[10] Carter SJ, Tattersall RS, Ramanan AV (2019). Macrophage activation syndrome in adults: recent advances in pathophysiology, diagnosis and treatment. Rheumatology.

[11] Sepulveda FE, de Saint BG (2017). Hemophagocytic syndrome: primary forms and predisposing conditions. Curr Opin Immunol.

[12] Triantafyllou E, Woollard KJ, McPhail MJW, Antoniades CG, Possamai LA (2018). The role of monocytes and macrophages in acute and acute-on-chronic liver failure. Front Immunol.

[13] Billiau AD, Roskams T, Van Damme-Lombaerts R, Matthys P, Wouters C (2005). Macrophage activation syndrome: characteristic findings on liver biopsy illustrating the key role of activated, IFN-γ-producing lymphocytes and IL-6- and TNF-α-producing macrophages. Blood.

[14] Ravelli A, Davì S, Minoia F, Martini A, Cron RQ (2015). Macrophage activation syndrome. Hematol Oncol Clin North Am.

[15] Griffin G, Shenoi S, Hughes GC (2020). Hemophagocytic lymphohistiocytosis: an update on pathogenesis, diagnosis, and therapy. Best Pract Res Clin Rheumatol.

[16] Khalifa A, Rockey DC (2020). The utility of liver biopsy in 2020. Curr Opin Gastroenterol.

[17] Transplant Hepatology Policies|ABIM.org. https://www.abim.org/certification/policies/internal-medicine-subspecialty-policies/transplant-hepatology.aspx. Accessed 25 July 2023.

[18] Tapper EB, Lok ASF (2017). Use of liver imaging and biopsy in clinical practice. N Engl J Med.

[19] Hagan M, Asrani SK, Talwalkar J (2015). Non-invasive assessment of liver fibrosis and prognosis. Expert Rev Gastroenterol Hepatol.

[20] Midia M, Odedra D, Shuster A, Midia R, Muir J (2019). Predictors of bleeding complications following percutaneous image-guided liver biopsy: a scoping review. Diagn Interv Radiol Ank Turk.

